# Combining patient visual timelines with deep learning to predict mortality

**DOI:** 10.1371/journal.pone.0220640

**Published:** 2019-07-31

**Authors:** Anoop Mayampurath, L. Nelson Sanchez-Pinto, Kyle A. Carey, Laura-Ruth Venable, Matthew Churpek

**Affiliations:** 1 Department of Pediatrics, University of Chicago, Chicago, IL, United States of America; 2 Division of Critical Care Medicine, Ann & Robert H. Lurie Children’s Hospital of Chicago, Chicago, IL, United States of America; 3 Department of Medicine, University of Chicago, Chicago, IL, United States of America; COMSATS University Islamabad, Wah Campus, PAKISTAN

## Abstract

**Background:**

Deep learning algorithms have achieved human-equivalent performance in image recognition. However, the majority of clinical data within electronic health records is inherently in a non-image format. Therefore, creating visual representations of clinical data could facilitate using cutting-edge deep learning models for predicting outcomes such as in-hospital mortality, while enabling clinician interpretability. The objective of this study was to develop a framework that first transforms longitudinal patient data into visual timelines and then utilizes deep learning to predict in-hospital mortality.

**Methods and findings:**

All adult consecutive patient admissions from 2008–2016 at a tertiary care center were included in this retrospective study. Two-dimensional visual representations for each patient were created with clinical variables on one dimension and time on the other. Predictors included vital signs, laboratory results, medications, interventions, nurse examinations, and diagnostic tests collected over the first 48 hours of the hospital stay. These visual timelines were utilized by a convolutional neural network with a recurrent layer model to predict in-hospital mortality. Seventy percent of the cohort was used for model derivation and 30% for independent validation. Of 115,825 hospital admissions, 2,926 (2.5%) suffered in-hospital mortality. Our model predicted in-hospital mortality significantly better than the Modified Early Warning Score (area under the receiver operating characteristic curve [AUC]: 0.91 vs. 0.76, *P* < 0.001) and the Sequential Organ Failure Assessment score (AUC: 0.91 vs. 0.57, *P* < 0.001) in the independent validation set. Class-activation heatmaps were utilized to highlight areas of the picture that were most important for making the prediction, thereby providing clinicians with insight into each individual patient’s prediction.

**Conclusions:**

We converted longitudinal patient data into visual timelines and applied a deep neural network for predicting in-hospital mortality more accurately than current standard clinical models, while allowing for interpretation. Our framework holds promise for predicting several important outcomes in clinical medicine.

## Introduction

Deep learning is a machine learning technique that has revolutionized predictive capabilities across many industries. An important application of deep learning is in image classification, where convolutional neural network (CNN) architectures have been shown to be at or above-par with human recognition [1–3]. These algorithms take images as inputs and learn generalizable patterns to identify objects and are commonly used in facial recognition software among other applications. They are also able to highlight areas within an image that contribute the most towards the prediction, thereby enabling better understanding of these models [4].

In medicine, CNNs have been used for image classification for diagnostic purposes such as identifying diabetic retinopathy in retinal fundus photographs [5,6], detecting lymph node metastases from tissue images [7], and detecting pulmonary disease from computed tomography images [8]. However, the majority of electronic health record (EHR) data elements, such as vital signs and laboratory results, are inherently represented in a non-image format. Developing a framework that converts longitudinal EHR data into visual representations would allow medical researchers to take advantage of powerful CNN algorithms and potentially improve accuracy in predicting patient outcomes over standard methods. In addition, this approach would also enable visual interpretability and recognition by clinicians, thereby creating a novel interface between these complex deep learning models and the human decision makers who use them [9].

The goal of this study was to develop a framework that first transforms granular EHR data into “visual timelines” and then inputs these images into a CNN with a recurrent layer architecture for prediction. The accuracy of this approach is investigated by using data from the initial 48 hours of inpatient stay for predicting in-hospital mortality. We compare prediction performances across other CNN architectures as well as a recurrent neural network (RNN). In addition, we demonstrate how to identify the area of the picture that was most important for making the prediction, thereby providing clinicians with insight into each individual patient’s prediction. The developed algorithm could be used to identify high-risk patients early in their hospitalization who may benefit from more aggressive care and/or goals of care discussions.

## Materials and methods

### Setting and study population

We conducted an observational cohort study of adult patient hospital admissions from November 2008 to January 2016 at the University of Chicago Medicine. Only patients admitted for at least 48 hours were included in the analysis. The study was approved by the University of Chicago Institutional Review Board (IRB#16–0608).

### Data sources

Clinical variables were collected from the electronic health record (Epic, Verona, WI) data, and patient demographics and discharge disposition (e.g., mortality) were determined from administrative data. All data elements were de-identified and extracted from the Clinical Research Data Warehouse (CRDW), maintained by the Center for Research Informatics (CRI) at the University of Chicago.

### Outcome and predictor variables

The primary outcome of the study was in-hospital mortality. Since we consider patients who were admitted for at least 48 hours, the primary outcome had to occur after the first 48 hours of hospital stay. Clinical variables used as predictors (i.e., model features) included all time-stamped vital signs, laboratory results, nurse examinations (e.g., Morse and Braden scores), diagnostics tests (e.g., chest x-rays), interventions (e.g., mechanical ventilation), and medications (grouped into medication type) that were collected during the first 48 hours of hospital stay. This timeframe was chosen in order to provide an early timepoint in a patient’s admission to make predictions, while also allowing time for determining if a patient is responding to therapy [10]. Patient clinical characteristics (demographics and co-morbidities from prior encounters) as well as location within the hospital were also included (see **S1 Table** for a full list of included variables). Data was blocked at hourly intervals with the most recent observation carried forward. Missing values for continuous variables (e.g. vital signs) were imputed by using location-specific medians.

**Fig 1:**
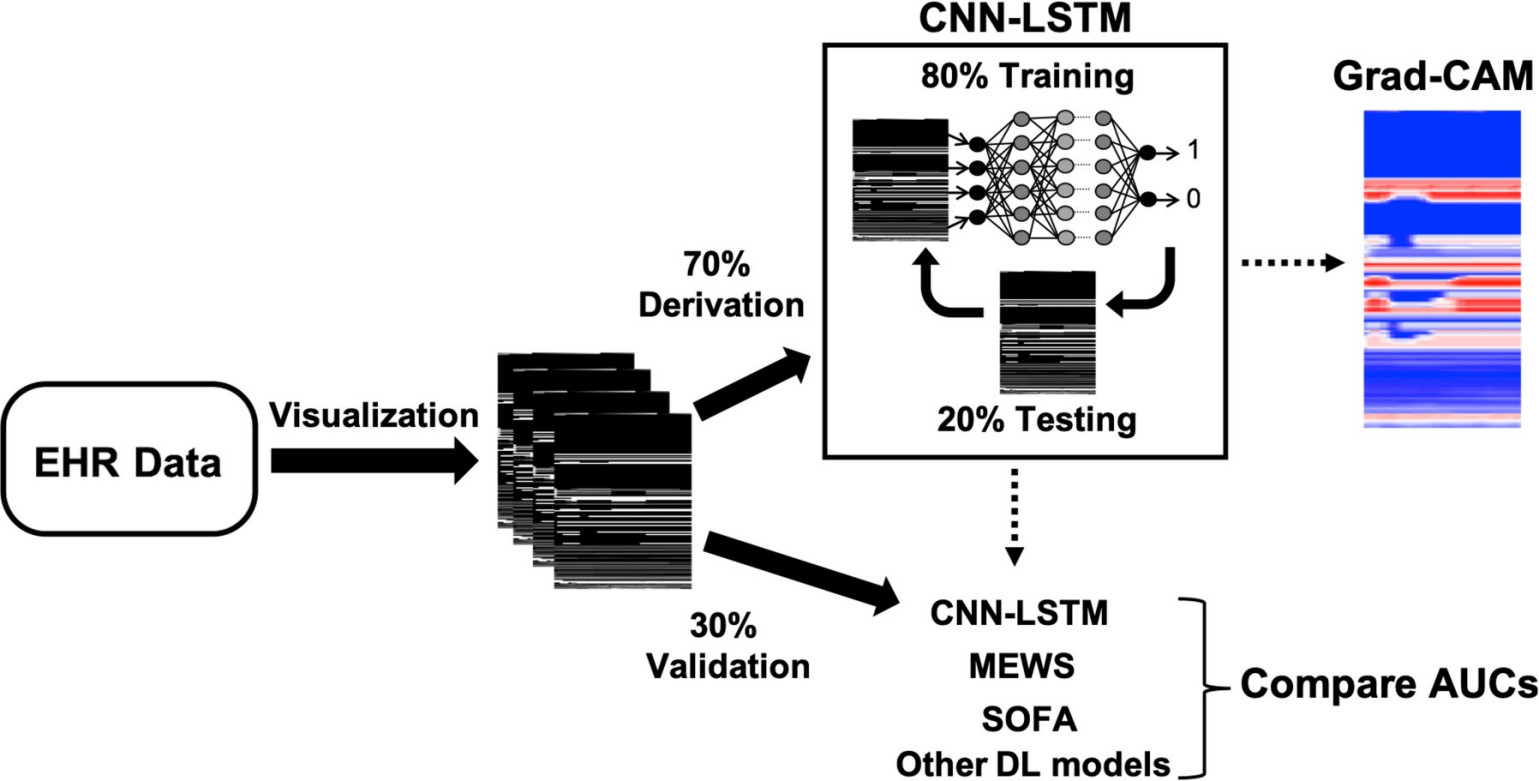
Diagrammatic representation of the study.

Two-dimensional visual representations for each patient were created with time on the x-dimension and predictor variables on the y-dimension (**Fig 1**). Each variable was normalized such that black/white indicates low/high values respectively for continuous variables and absence/presence respectively for binary variables. The final feature matrix for a single patient admission was 156 variables x 48 time points (i.e., one time point per hour since admission).

### Feature engineering

**Fig 2:**
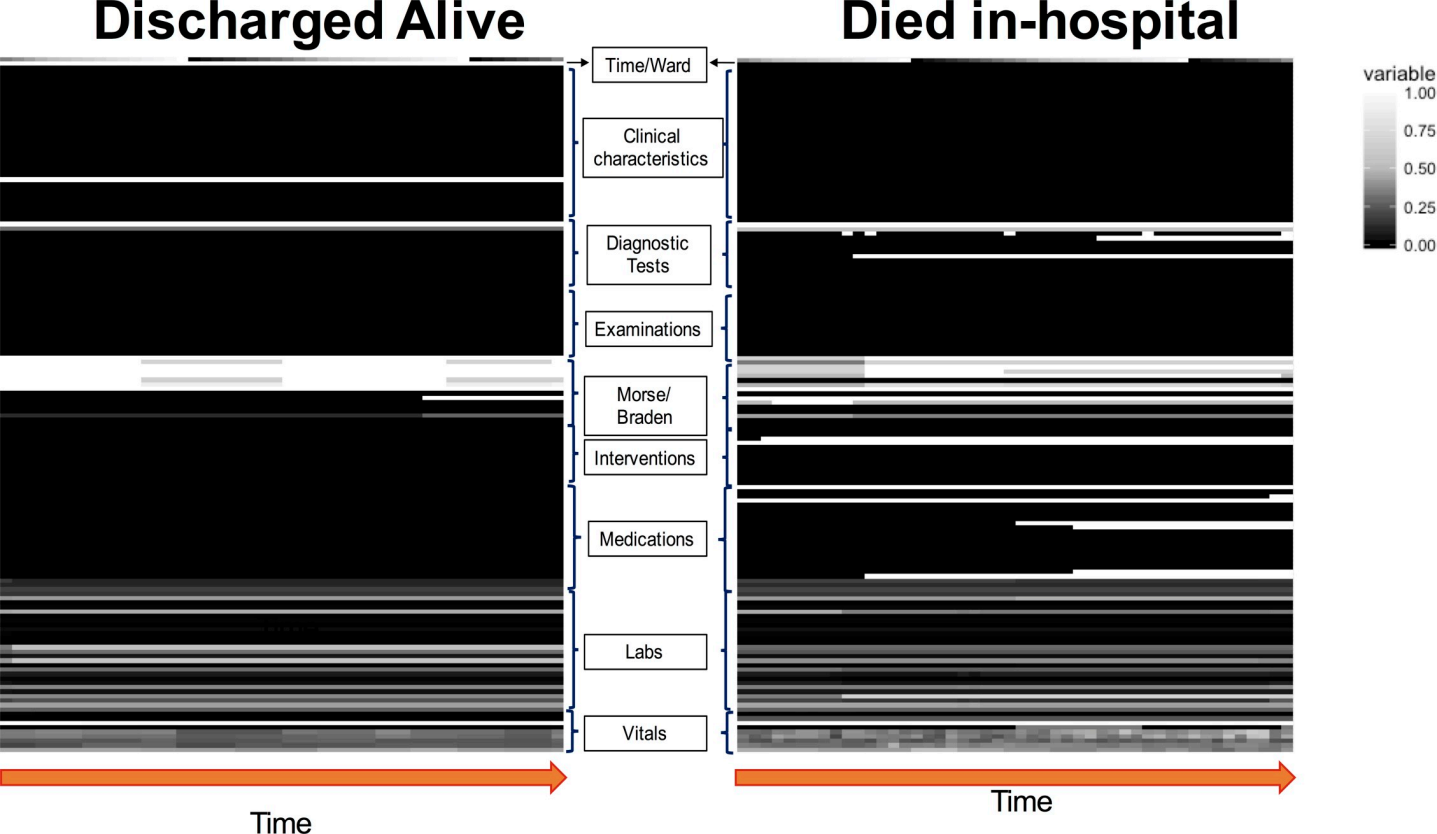
Visual timelines of two patients, one who was discharged alive (left) and one died during hospital stay (right). Time from admission (0–48 hours) is on the x-axis and variables grouped by category are on the y-axis. Continuous variables (e.g. temperature) are normalized so that black indicates minimum and white indicates maximum values. Binary variables (e.g. vasopressor medication) are represented so that black indicates absence and white indicates presence of the variable. As shown, the visual timeline of the patient who died depicts increased activity in the intervention and medication areas as well as multiple changes to vital sign readings as compared to the visual timeline of the patient who was survived the hospitalization.

We considered three ways of variable orderings for the visual timelines: (1) standard ordering, where the variables are arranged by type of variable but with no specific method used to order variables within a group (as showing in **Fig 1**), (2) within-group cluster ordering, where variables are clustered and arranged spatially by correlations within each group, and (3) across-group cluster ordering, where variables are clustered across all groups and spatially arranged by correlation regardless of variable type. The R package corrplot was used to determine the ordering for methods 2 and 3.

We further considered two normalization schemes. In the first normalization method (called “min-max”), variables were normalized to a minimum-zero maximum-one scale (as shown in **Fig 1**) depending on type; continuous variables (e.g., respiratory rate) were normalized to a minimum-zero and maximum-one scale and binary variables (e.g., medication) were normalized to indicate absence (zero) or presence (one) of the variable at each time point. For the second normalization method (called “custom”), each continuous variable was normalized zero-to-one so that zero is considered normal and one is considered abnormal for that variable. For example, in FiO2, normalized values closer to one will indicate higher delivered FiO2 values that are typically associated with abnormal patient clinical status, while for variables like urine output, the inverse is done, where values close to one indicate low urine output, generally associated with poor outcomes. Finally, variables that can be abnormal at both low and high values (e.g., heart rate) were normalized to mean zero so that being close to one indicates values that are away from the mean (i.e., either higher or lower than the mean value for that variable). Binary variables (e.g., medications) were retained as absence or normal (indicated by 0) or presence or abnormal (indicated by 1) in the custom normalization scheme.

### Deep learning model

We utilized several deep learning algorithms in this study. Our first convolutional neural network (called Standard-CNN here) is a standard architecture used in classifying handwritten digits, such as those in the MNIST database. We also considered a standard recurrent neural network (called RNN here) as well as the more advanced Inception-v3 deep CNN architecture (called Deep-CNN here), an advanced deep neural network that has shown to be highly accurate in large-scale image classification tasks [11,12]. Finally, we also utilized a variation of Standard-CNN with a recurrent layer (called CNN-RL here), which combines both spatial and temporal information in visual timelines for prediction. Details on each model’s architecture and hyperparameters are given in **S2 Table**.

**Fig 1** depicts our machine learning derivation and testing framework. Briefly, we split our patient population randomly into 70% for model derivation and 30% for independent validation. The deep learning models were derived using 80% training split of the derivation data with hyperparameter optimization based on model performance on the remaining 20% testing split of the derivation data. Final predictions were performed on the hold-out independent validation dataset and the area under the receiver operating characteristic curve (AUC) was compared between all deep learning models and two traditional risk stratification scores: the Modified Early Warning Score (MEWS) [13] and the Sequential Organ Failure Assessment (SOFA) score [14]. Briefly, the MEWS score is a risk stratification tool used by care personnel to recognize deterioration in hospitalized ward patients. The scoring is based on vital signs and nurse-documented patient consciousness. The SOFA score is used to assess risk of organ failure, acuity, and mortality in patients and is sequentially based on worst values of vital signs during a time period. We used each patient’s highest MEWS and SOFA score within the first 48 hours of hospitalization to determine their performance in predicting mortality post 48 hours of hospital admission.

Class-activation maps were created for CNN-RL using the Grad-CAM algorithm in order to highlight areas within the image important to the prediction for an individual patient [4]. Class activation map visualization generally involves a picture representation of a 2D grid of scores associated with a specific class label. The scores are calculated for every pixel in the image and is an indication of how important each image location is with respect to the class label. The Grad-CAM algorithm in particular does this by using the class label-specific gradient information at the final convolutional layer of the CNN to produce a coarse and localized heatmap of the regions of the image important to predicting the class. We created attention-based heatmap representations of each patient’s visual timeline that utilized the Grad-CAM algorithm to color-code areas important to prediction of the outcome for that patient. Higher attention areas are coded towards red indicating importance towards predicting the outcome by the CNN-RL.

Analyses were performed using R version 3.3 (R Project for Statistical Computing), with two-sided *P <* .05 denoting statistical significance. The keras library version 2.1.5 (http://keras.rstudio.com) was utilized for building the deep learning models on a Dual NVIDIA Quadro P5000 graphics processing unit.

## Results

### Study population

Among 115,825 hospital admissions, a total of 2,926 (2.5%) died during their hospital stay. Patients who died in-hospital were older (mean age 63 years vs 55 years, *P* < 0.001), less likely to be female (47% vs. 57%, *P <* 0.001) or black (48% vs. 54%, *P <* 0.001) as compared to patients who were discharged alive (**Table 1**). They were also more likely to be admitted to the intensive care unit (ICU; 25% vs. 5%, *P* < 0.001) and less likely to be admitted to areas other than ward, ICU, or the emergency department (e.g., procedural areas, 4% vs. 23%, *P* < 0.001). Finally, patients with in-hospital mortality had a higher median length-of-stay (9 vs. 5 days, *P* < 0.001) as compared to patients discharged alive.

**Table 1:** Comparison of characteristics between patients who experienced in-hospital mortality and those who were discharged alive.

Attributes	Patient admissions that had in-hospital mortality (n = 2,926)	Patient admissions that were discharged alive (n = 112,899)
Age, mean(SD), yrs	63 (15)*	55 (19)
Female sex, n (%)	1,377 (47)*	64,600 (57)
Race Black, n (%)	1,394 (48)*	60,732 (54)
Admission Location		
Ward, n (%)	807 (28)*	34,850 (31)
ED, n (%)	1,272 (43)*	46,541 (41)
ICU, n (%)	744 (25)*	6,002 (5)
Other, n (%)	103 (4)*	25,506 (23)
LOS, days, median (IQR)	9 (5,17)*	5 (3, 7)

*P < 0.001 compared to patients discharged alive.

IQR: Inter-quantile range

### Visual timelines

**Fig 2** depicts the comparison of visual timelines between a patient who died (right) versus a patient who was discharge alive (left) using standard ordering and min-max normalization. As shown, the visual timeline of the patient who died reveals multiple changes in vital signs and more “activity” in the labs, interventions, and medication areas as compared to the visual timeline of the patient discharged alive. To determine the most discriminating variables, we created a differential image by subtracting the average visual timeline for patients who died in-hospital from the average visual timeline of patients who were discharged alive (see **S1 Fig**). Variables indicating maximum difference are depicted in white and include subsets of vital signs, medications, interventions, Morse and Braden scores, and diagnostic tests.

### Model performance

AUCs for mortality prediction are shown in **Table 2.** Our model (CNN-RL) predicted in-hospital mortality using data from the first 48 hours of patient admission more accurately than either MEWS (0.91 vs. 0.76, *P* < 0.001) or SOFA (0.91 vs. 0.57, *P* < 0.001), with death occurring a median of seven days after the last time point of data that was used to derive the model. Further, the CNN-RL predicted the primary outcome better than the Standard-CNN (0.91 vs. 0.87, *P* < 0.001), the RNN (0.91 vs. 0.89, *P* = 0.003) and the Deep-CNN (0.91 vs. 0.90, *P* = 0.025). The CNN-RL model had a higher area under the precision recall curve (AUPR = 0.33) when compared to MEWS (AUPR = 0.10), SOFA (AUPR = 0.05), and Standard CNN (AUPR = 0.24). The AUPR of the CNN-RL model was comparable to RNN (AUPR = 0.32), and Deep-CNN (AUPR = 0.34).

**Table 2:** Model discrimination for predicting mortality on the test dataset (n = 34,747 admissions).

Model	AUC (95% CI)	*P*-value (compared to CNN-RL)
SOFA	0.57 (0.55, 0.59)	<0.001
MEWS	0.76 (0.74, 0.78)	<0.001
Standard-CNN	0.87 (0.85, 0.88)	<0.001
RNN	0.89 (0.88, 0.91)	0.003
Deep-CNN	0.90 (0.89, 0.91)	0.025
CNN-RL	0.91 (0.90, 0.92)	-

SOFA: Sequential Organ Failure Assessment score

MEWS: Modified Early Warning Score

CNN: Convolutional Neural Network

RNN: Recurrent Neural Network

RL: Recurrent Layer

AUC: Area Under the receiver operating characteristic Curve

CI: Confidence Interval

**S3 Table** depicts the AUCs for predicting mortality on the test dataset for different visual timeline configurations. Apart from a slight drop in AUCs using the across-group ordering scheme, the AUCs stayed consistently high for all other visual timeline configurations. Prediction accuracy for the CNN-RL model was comparable between patients located in the ICU at the end of the first 48 hours and patients on the ward at 48 hours (AUC 0.86, 95% CI: 0.85–0.88 vs. 0.87, 95% CI 0.86–0.89, **S4 Table**). Single pixel perturbations in the test dataset, by randomly zeroing out a pixel within each patient’s visual timeline, did not impact predictive performance (AUC 0.91, 95% CI 0.90–0.92 vs. 0.91, 95% CI 0.90–0.92). The Standard-CNN and the CNN-RL models took one minute per epoch on average to train, while the Deep-CNN and the RNN model took on average approximately three minutes per epoch.

**Fig 3:**
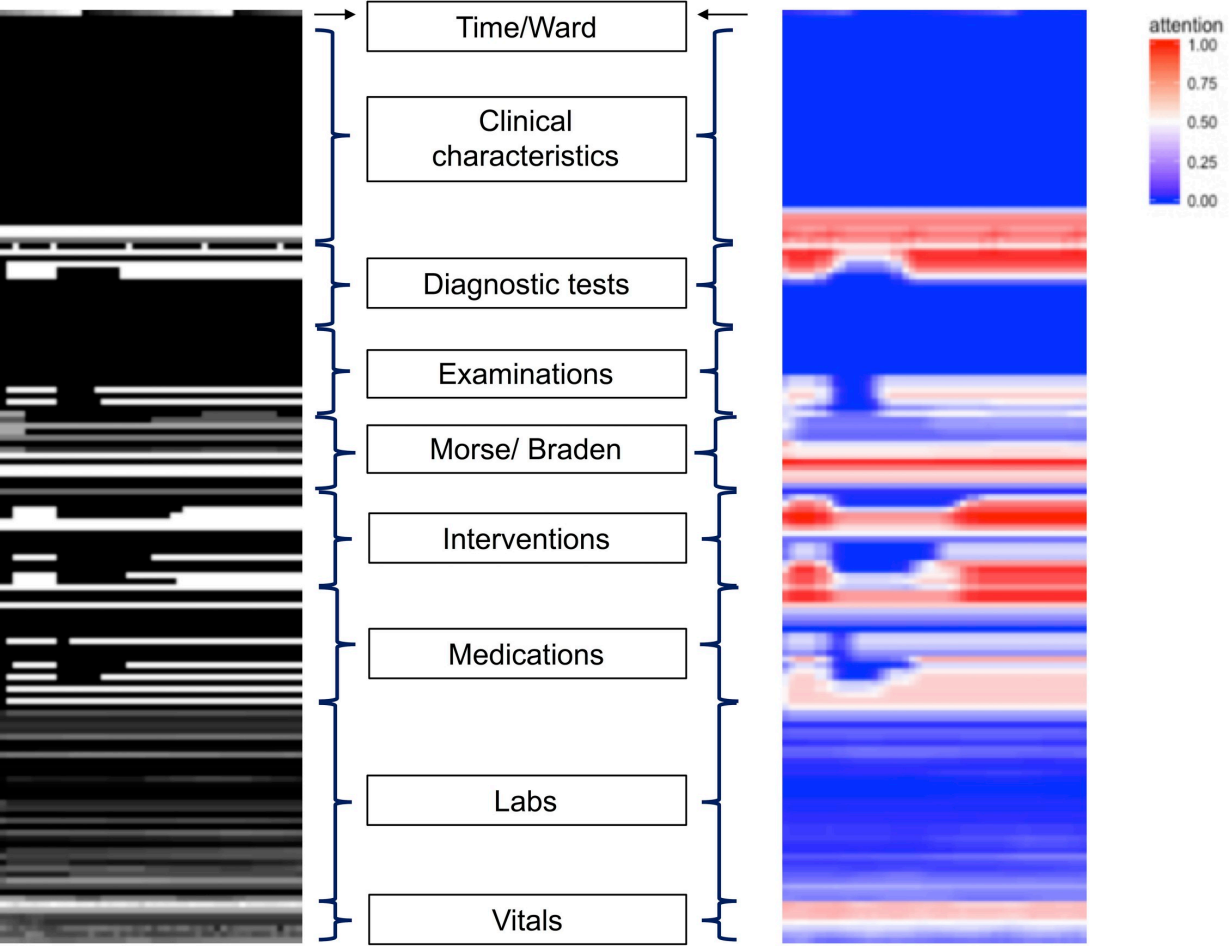
Original (left) and Grad-CAM attention-based heatmap (right) derived for the CNN-RL model for a min-max normalized standard ordering visual timeline of a test patient who died in-hospital. As can be seen, the area of interventions and diagnostic testing have been highlighted as the most contributing areas for predicting a high probability of mortality for this patient.

**Fig 3** depicts an example visual timeline with an adjoining class activation heatmap acquired using Grad-CAM. The heatmap highlights the areas of the image that were most important (i.e., garnered most attention) for the CNN-RL model when making its prediction. For this patient who died in-hospital, the features that most contributed to the decision-making were early and late interventions, diagnostic tests, and certain Morse and Braden scores within the first 48 hours. The medications, other Morse and Braden exam scores, and vitals areas within the picture were the next most-contributory features, while areas of the picture that pertained to clinical characteristics and labs were the least important for the Deep-CNN model when making the prediction.

## Discussion

In this study, we developed a framework that converts clinical data into visual representations in order to facilitate the application of deep learning. Our main finding is that this framework can capture both repeatable patterns in the data, through use of a CNN architecture, as well as the ordering of these patterns, through a recurrent layer. We also found that, when applied to predicting in-hospital mortality using data from only the first 48 hours of admission, our architecture outperformed standard clinical models in a cohort of over 100,000 patient admissions. In addition, our model outperformed simple and complex convolutional neural network models as well as a recurrent neural network model. Our model predicted mortality, on average, a week in advance of the event, and required almost no feature engineering (i.e., manual transformation of variables prior to training). In addition, our study finds that deep learning model can identify specific areas within the visual timeline that were most contributory to the prediction, facilitating the interpretation of the model by clinicians.

Prior studies have utilized deep learning to predict different outcomes using data natively captured as images (e.g., radiology images). For example, CNNs have been shown to have high sensitivity and specificity for detecting referable diabetic retinopathy using retinal fundus photographs [5, 6]. In a study aimed at detecting lymph node metastases in tissue sections in women with breast cancer, seven deep learning algorithms outperformed a panel of eleven pathologists [7]. CNNs have also been utilized to detect and stage chronic obstructive pulmonary disease from computerized tomography images of smokers [8]. Our study adds to this body of work by transforming non-image longitudinal EHR data into an image format and then applying deep learning to predict in-hospital mortality.

Deep learning algorithms have also been applied to structured and unstructured EHR data in a non-image format (see [15] for a comprehensive review). Recent studies have demonstrated the utility in exploring different patient representations from notes [16] and medical codes, such as diagnoses, medications, and procedures [17–19]. Other studies have reported high performance for predicting all-cause mortality within a year given data from the prior year [20], predicting diagnostic onset of 133 conditions using lab tests [21], and predicting interventions in ICU patients [22]. A recent study by Rajkomar et al. used advanced deep learning model architectures to predict mortality and other outcomes, including unplanned readmissions and prolonged length of stay, using longitudinal data from the first 24 hours of patient admission [23]. Our model distinguishes itself from this prior work by using image representations of structured EHR data combined with a recurrent layer to incorporate the temporal aspects of the data, while additionally providing a way to visually determine what part of the image was important for prediction, which could allow a higher degree of clinician interpretability. Additionally, our study considers patients who have stayed in hospital for at least 48 hours, thereby incorporating a patient’s to response to therapy.

While the use of deep learning algorithms has provided encouraging results with high accuracy at predicting clinical outcomes, one issue that remains relatively unaddressed is the interpretability trade-off. Generally, the goal of prediction models is to uncover the relationship between predictors and outcomes in existing data in order to predict outcomes in future data. This relationship can be modeled using relatively simple and inflexible functions like linear regression, which provides coefficients for each predictor in the model and are relatively straightforward to interpret. However, unless the underlying relationship between the predictors and the outcomes is mostly linear (which is rare in medicine) this approach will only achieve modest accuracy, as has been demonstrated in prior work [24,25]. On the other hand, many machine learning algorithms can generate flexible functions that can uncover the often-complex relationship between predictors and the outcomes and therefore achieve high accuracy. However, this is done at the expense of model interpretability, given that these complex functions are hard for humans to understand, and are often referred to as “black boxes” [9]. Our approach takes advantage of the highly accurate deep learning algorithms while providing an interpretable visual output. For example, patient visual timelines can be used as a visible indicator of a patient’s clinical profile during a time period by aggregating disparate variables such as laboratory results and vital signs into a single pane, as illustrated in Fig 1. Further, highlighting which part of the visual timeline is most important to the prediction of patient’s risk through Grad-CAM can enable clinicians to zoom into certain regions to see abnormal readings in a particular vital sign or the ordering of a particular diagnostic tool. This visual interface would thus not only allow clinicians better understand the algorithm’s predictions, but also enable their pattern recognition abilities and potentially open the door to new ways of interacting with complex and voluminous longitudinal data in the clinical setting. Future work will focus on testing the usability of patient visual timelines in an interactive manner as well as exploration of novel visualization tools such as Activation Atlas [26] for a deeper interpretation.

Our work builds on that of Ledbetter and Azcon, who applied a CNN framework to patients in a pediatric ICU [27]. Our study has expanded their approach by including a recurrent layer to incorporate the temporal aspects of the data, predicting mortality in a complex and diverse population of all hospital patients, exploring various mechanisms of creating images, and illustrating methods to increase the interpretability of these models using the developed visualizations. In particular, we found that predictive performance drops when variables are not grouped by type, suggesting that this arrangement of clinical variables is important. The exact ordering of variables within each group as well as the normalization method does not impact prediction accuracy, validating that deep learning methods do not require extensive feature engineering.

Our model has the potential to be used for clinical care in several ways. First, the visual timelines provide clinicians with a way to interact with complex and voluminous longitudinal data. Second, the visual timeline can be used to interface with the output of the deep learning algorithm by highlighting areas of the image that most contribute to the prediction. Finally, from a clinical decision-making standpoint, our prediction model can identify patients at risk of death a week in advance, which is early enough to warrant increased patient monitoring, more aggressive care, or prompt further goals-of-care discussions.

There are several limitations to our study. First, this study was performed at a single academic medical center, so our model may not be generalizable to other hospitals. Future work is needed to determine the accuracy of the model in other clinical settings. Second, we have only highlighted one use-case in predicting in-hospital mortality. The efficacy of our framework for predicting other patient outcomes needs to be explored. Additionally, our framework addresses missing data by carrying forward last known observation and median imputing if no prior observations are present. As per prior work, our framework is potentially vulnerable to artificial perturbations in visual timelines [28]. However, we noticed no change in performance when randomly zeroing out single pixels within each visual timeline in the test dataset. Exploration of alternate architectures that address the problem of image perturbation will be an area of future work. Finally, there are a wide variety of machine learning models developed for predicting patient outcomes that need to be compared to our deep learning framework. However, our framework offers a degree of interpretability through the use of class-activation maps to highlight variables of importance for prediction, which is not available for most other algorithm types.

In conclusion, we developed a framework that uniquely represents a patient’s EHR-based clinical data in a visual format and uses a deep learning neural network model for predicting in-hospital mortality using information from the first 48 hours of hospital stay. Our framework not only shows the feasibility of using deep learning in clinical data, but also provides a way for early identification of patients at-risk for death and for clinicians to visually interact with complex data while interpreting the output of the deep learning prediction model.

## Supporting information

S1 Fig Variable importance. Variables of importance in distinguishing patients who died from patients who were discharged alive. This differential picture was obtained by subtracting the average visual timeline of patients who died from the average visual timeline of patients who were discharged alive. Whiter areas indicate variables of maximum difference between the two groups of patients, and black indicates variables that were the similar.
(TIFF)

S1 Table Complete list of variables included in the full model. (DOCX)

S2 Table Architectures and hyperparameters of deep learning models used in this study. (DOCX)

S3 Table Performance with different variable ordering and normalization. Comparison of AUCs for CNN-RL with different ordering of variables as well as different normalization schemes. Values shown are AUCs (95% CI).
(DOCX)

S4 Table Performance with patient location. CNN-RL AUC comparison between patient location at 48 hours (ward vs. ICU) on a test dataset of 34,747 patient admissions.
(DOCX)
