# Supplementary material for: Combining patient visual timelines with deep learning to predict mortality
Source: PLoS One. 2019 Jul 31;14(7):e0220640. doi: 10.1371/journal.pone.0220640 (PMC6668841; doi:10.1371/journal.pone.0220640)
Supplement: S1 Table — (DOCX) [file pone.0220640.s002.docx]

**S1 Table. Complete list of variables included in the full model**

| Patient Characteristics | Demographics: Age, Sex, Race | Continuous - age  Binary  – sex (male vs. female), race (black vs. other) |
| --- | --- | --- |
|  | Prior conditions: Prior cardiac arrest, Prior admission within 90 days | Binary |
| Time | Hour of day | Continuous |
| Location | Current location (ICU vs. ED vs. other) | Categorical |
| Vital signs | Temperature (C°), Heart Rate, Respiratory Rate, Systolic Blood Pressure (SBP), Diastolic Blood Pressure (DBP), O2 Saturation, FiO2, AVPU | Continuous |
| Laboratory values | Basic Metabolic Panel [BMP]: Sodium, Potassium, Bicarbonate (CO2), Anion Gap, Glucose, Calcium, Blood Urea Nitrogen (BUN), Serum Creatinine (SCr), BUN/SCr ratio, Phosphate | Continuous |
|  | Liver Function Test [LFT]: Total Protein, Albumin, Total Bilirubin, AST (SGOT), Alkaline Phosphatase |  |
|  | Complete Blood Count [CBC]: White Blood Cells (WBC), Hemoglobin, Platelet Count |  |
|  | Other labs: Lactate, Troponin, pH, Ketones, Chloride, International Normalized Ration (INR), Lipase, Mean Corpuscular Volume (MCV), Partial pressure carbon dioxide (PaCO2), Partial pressure oxygen (PaO2), Partial Thromboplastin Time (PTT), Red cell Distribution Width (RDW) |  |
| Interventions | Interventions: Dialysis, IV bolus (0.9% Sodium Chloride, Lactated Ringers), Albumin (5% or 25%), Using Ventilator, Using BiPAP, Using CPAP, Using HFNC, Using Suction | Binary |
|  | Transfusions: Red Blood Cell (RBC) transfusion, Fresh Frozen Plasma (FFP) transfusion, Platelet transfusion, Cryoprecipitate transfusion | Binary |
| Medications | Nebulizer treatments, IV/SC Hypoglycemics, PO Hypoglycemics, Drip Hypoglycemics, Lactulose, IV AV Nodal Blockers, PO AV Nodal Blockers, IV Antiarrhythmic, PO Antiarrhythmic, Anti Seizures, IV Anticoagulants, IV Steroids, PO Steroid, IV Immunotherapy, PO Immunotherapy, IV Antipsychotics, PO Antipsychotics, Sedative Drips, IV Benzodiazepine, PO Benzodiazepine, Vasopressors, Inotropes, IV Diuretics, PO Diuretics, Antibiotics | Binary |
| Examinations | Cardiac Rhythm: Paced, Atrial Fibrillation, Atrial flutter, Using?, Superventricular SVT, VT, VF, Asystole, Heart Block, Junctional Rhythm | Binary |
| Morse/Braden | Summed score and score components | Continuous |
| Diagnostics/Urinary | Diagnostics: EKG, TTE, Chest X-ray, Abdomen X-ray, CT Scan (head, neck, chest, and abdomen), Ultrasound, Blood Culture Order | Binary |
|  | Urinary Bag: Urine Output, Foley catheter placed | Binary |

FiO2-fraction of inspired oxygen, AVPU- Alert, Voice, Pain, Unresponsive Scale; BiPAP-Bilevel Positive Airway Pressure, CPAP- Continuous Positive Airway Pressure, HFNC-High Flow Nasal Canula, EKG- Electrocardiogram, TTE-Transthoracic Echocardiography, CT- Computed Tomography
