# Supplementary material for: Combining patient visual timelines with deep learning to predict mortality
Source: PLoS One. 2019 Jul 31;14(7):e0220640. doi: 10.1371/journal.pone.0220640 (PMC6668841; doi:10.1371/journal.pone.0220640)
Supplement: S2 Table — (DOCX) [file pone.0220640.s003.docx]

**S2 Table**: Architectures and hyperparameters of deep learning models used in this study.

| Model | Architecture | Hyperparameters |
| --- | --- | --- |
| Standard-CNN | Convolutional Layer (32) -> Convolutional Layer (64) ->  Max Pooling -> Dropout (25%) -> Flatten -> Dense (128) -> Dropout (50%) -> Softmax. | Relu Activation, Batch Size 128, RMS Prop Optimizer, Learning rate 1E-3, Decay 0, 1:30 Class Weight, 20 epochs with early stopping (Min. Delta 0.01, Patience 3) |
| RNN | LSTM (128) -> Dropout (20%) -> LSTM (128, Relu activation) -> Dropout (10%) -> Dense (32) -> Dropout (20%) -> Softmax | Relu activation, Batch Size 128, Adam Optimizer, Learning rate 1E-4, Decay 1E-06, 1:30 Class Weight, 20 epochs with early stopping (Min. Delta 0.01, Patience 3) |
| Deep-CNN | Inception V3 architecture [11] | Relu activation, Batch Size 128, RMS Prop Optimizer, Learning rate 1E-3, Decay 0, 1:30 Class Weight, 20 epochs with early stopping (Min. Delta 0.01, Patience 3) |
| CNN-RL | Convolutional Layer (32) -> Convolutional Layer (64) ->  Max Pooling -> Dropout (25%) -> Conv_LSTM (8) -> Flatten -> Dense (128) -> Dropout (25%) -> Softmax | Relu activation, Batch Size 128, RMS Prop Optimizer, Learning rate 1E-3, Decay 1E-06, 1:30 Class Weight, 20 epochs with early stopping (Min. Delta 0.01, Patience 3) |
