# Supplementary material for: Combining patient visual timelines with deep learning to predict mortality
Source: PLoS One. 2019 Jul 31;14(7):e0220640. doi: 10.1371/journal.pone.0220640 (PMC6668841; doi:10.1371/journal.pone.0220640)
Supplement: S3 Table — Comparison of AUCs for CNN-RL with different ordering of variables as well as different normalization schemes. Values shown are AUCs (95% CI). (DOCX) [file pone.0220640.s004.docx]

**S3 Table. Performance with different variable ordering and normalization.** Comparison of AUCs for CNN-RL with different ordering of variables as well as different normalization schemes. Values shown are AUCs (95% CI).

|  | Standard  Ordering | Within-group Ordering | Across-group  Ordering |
| --- | --- | --- | --- |
| Min-max  normalization | 0.91 (0.90, 0.92) | 0.90 (0.89, 0.91) | 0.89 (0.88, 0.90) |
| Custom  normalization | 0.90 (0.89, 0.91) | 0.89 (0.88, 0.90) | 0.89 (0.88, 0.90) |
