# Supplementary material for: Combining patient visual timelines with deep learning to predict mortality
Source: PLoS One. 2019 Jul 31;14(7):e0220640. doi: 10.1371/journal.pone.0220640 (PMC6668841; doi:10.1371/journal.pone.0220640)
Supplement: S4 Table — CNN-RL AUC comparison between patient location at 48 hours (ward vs. ICU) on a test dataset of 34,747 patient admissions. (DOCX) [file pone.0220640.s005.docx]

**S4 Table. Performance with patient location.** CNN-RL AUC comparison between patient location at 48 hours (ward vs. ICU) on a test dataset of 34,747 patient admissions.

| Location | Patients who died (n, %) | AUC (95% CI) |
| --- | --- | --- |
| All locations  (n=34,747) | 885 (2.5) | 0.91 (0.90, 0.92) |
| Ward  (n=29,373) | 358 (1.2) | 0.87 (0.86, 0.89) |
| ICU (n=4,746) | 519 (10.9) | 0.86 (0.85, 0.88) |
